# Cellular and Structural Basis of Synthesis of the Unique Intermediate Dehydro-F_420_-0 in Mycobacteria

**DOI:** 10.1128/mSystems.00389-20

**Published:** 2020-05-19

**Authors:** Rhys Grinter, Blair Ney, Rajini Brammananth, Christopher K. Barlow, Paul R. F. Cordero, David L. Gillett, Thierry Izoré, Max J. Cryle, Liam K. Harold, Gregory M. Cook, George Taiaroa, Deborah A. Williamson, Andrew C. Warden, John G. Oakeshott, Matthew C. Taylor, Paul K. Crellin, Colin J. Jackson, Ralf B. Schittenhelm, Ross L. Coppel, Chris Greening

**Affiliations:** a School of Biological Sciences, Monash University, Clayton, VIC, Australia; b Department of Microbiology, Monash Biomedicine Discovery Institute, Monash University, Clayton, VIC, Australia; c CSIRO Land & Water, Canberra, ACT, Australia; d Research School of Chemistry, Australian National University, Canberra, ACT, Australia; e Department of Biochemistry, Monash Biomedicine Discovery Institute, Monash University, Clayton, VIC, Australia; f Monash Proteomics & Metabolomics Facility, Monash Biomedicine Discovery Institute, Monash University, Clayton, VIC, Australia; g Department of Microbiology and Immunology, University of Otago, Dunedin, New Zealand; h Peter Doherty Institute for Infection and Immunity, University of Melbourne, Melbourne, VIC, Australia; University of California San Diego

**Keywords:** cofactor biosynthesis, deazaflavin, F_420_, *Mycobacterium*, *Mycobacterium smegmatis*, structural biology

## Abstract

F_420_ is a low-potential redox cofactor used by diverse bacteria and archaea. In mycobacteria, this cofactor has multiple roles, including adaptation to redox stress, cell wall biosynthesis, and activation of the clinical antitubercular prodrugs pretomanid and delamanid. A recent biochemical study proposed a revised biosynthesis pathway for F_420_ in mycobacteria; it was suggested that phosphoenolpyruvate served as a metabolic precursor for this pathway, rather than 2-phospholactate as long proposed, but these findings were subsequently challenged. In this work, we combined metabolomic, genetic, and structural analyses to resolve these discrepancies and determine the basis of F_420_ biosynthesis in mycobacterial cells. We show that, in whole cells of Mycobacterium smegmatis, phosphoenolpyruvate rather than 2-phospholactate stimulates F_420_ biosynthesis. Analysis of F_420_ biosynthesis intermediates present in M. smegmatis cells harboring genetic deletions at each step of the biosynthetic pathway confirmed that phosphoenolpyruvate is then used to produce the novel precursor compound dehydro-F_420_-0. To determine the structural basis of dehydro-F_420_-0 production, we solved high-resolution crystal structures of the enzyme responsible (FbiA) in apo-, substrate-, and product-bound forms. These data show the essential role of a single divalent cation in coordinating the catalytic precomplex of this enzyme and demonstrate that dehydro-F_420_-0 synthesis occurs through a direct substrate transfer mechanism. Together, these findings resolve the biosynthetic pathway of F_420_ in mycobacteria and have significant implications for understanding the emergence of antitubercular prodrug resistance.

**IMPORTANCE** Mycobacteria are major environmental microorganisms and cause many significant diseases, including tuberculosis. Mycobacteria make an unusual vitamin-like compound, F_420_, and use it to both persist during stress and resist antibiotic treatment. Understanding how mycobacteria make F_420_ is important, as this process can be targeted to create new drugs to combat infections like tuberculosis. In this study, we show that mycobacteria make F_420_ in a way that is different from other bacteria. We studied the molecular machinery that mycobacteria use to make F_420_, determining the chemical mechanism for this process and identifying a novel chemical intermediate. These findings also have clinical relevance, given that two new prodrugs for tuberculosis treatment are activated by F_420_.

## Abstract (precis)

Mycobacteria are major environmental microorganisms and cause many significant diseases, including tuberculosis. Mycobacteria make an unusual vitamin-like compound, F_420_, and use it to both persist during stress and resist antibiotic treatment. Understanding how mycobacteria make F_420_ is important, as this process can be targeted to create new drugs to combat infections like tuberculosis. In this study, we show that mycobacteria make F_420_ in a way that is different from other bacteria. We studied the molecular machinery that mycobacteria use to make F_420_, determining the chemical mechanism for this process and identifying a novel chemical intermediate. These findings also have clinical relevance, given that two new prodrugs for tuberculosis treatment are activated by F_420_.

## INTRODUCTION

Factor 420 (F_420_) is a deazaflavin cofactor that mediates diverse redox reactions in bacteria and archaea (1). Chemically, F_420_ consists of a redox-active deazaflavin headgroup (derived from the chromophore Fo) that is conjugated to a variable-length polyglutamate tail via a phosphoester linkage (2). While the Fo headgroup of F_420_ superficially resembles flavins (e.g., flavin adenine dinucleotide [FAD], flavin mononucleotide [FMN]), three chemical substitutions in the isoalloxazine ring give it distinct chemical properties more reminiscent of nicotinamides (e.g., NADH, NADPH) (1). These properties include a low standard potential (−350 mV) and obligate two-electron (hydride) transfer chemistry (3, 4). The electrochemical properties of F_420_ make it ideal to reduce a wide range of otherwise recalcitrant organic compounds (5–7). Diverse microorganisms are known to synthesize F_420_, but the compound is best characterized for its roles in methanogenesis in archaea, antibiotic biosynthesis in streptomycetes, and metabolic adaptation of mycobacteria (1, 8–11). In mycobacteria, F_420_ is involved in a plethora of processes: central carbon metabolism, cell wall synthesis, recovery from dormancy, resistance to oxidative stress, and inactivation of certain bactericidal agents (7, 12–14). In the human pathogen Mycobacterium tuberculosis, F_420_ is also critical for the reductive activation of the newly approved clinical antitubercular prodrugs pretomanid and delamanid (15–17).

Following the elucidation of the chemical structure of F_420_ in the 1970s, the F_420_ biosynthesis pathway in archaea was determined through a combination of *in situ* biochemistry and recombinant protein analysis (1, 2). Described briefly, the deazaflavin fluorophore Fo is synthesized through condensation of 5-amino-6-ribitylamino-2,4(1H,3H)-pyrimidinedione and l-tyrosine by the *S*-adenosyl-l-methionine (SAM)-radical enzymes CofG and CofH (18). The putative enzyme CofB synthesizes 2-phospholactate (2PL), which links Fo to the glutamate tail of mature F_420_ (19). Subsequently, the nucleotide transferase CofC condenses 2PL with GTP to form the reactive intermediate l-lactyl-2-diphospho-5ʹ-guanosine (LPPG) (20). The phosphotransferase CofD then transfers 2PL from LPPG to Fo, leading to the formation of F_420_-0 (i.e., F_420_ with no glutamate tail) (21). Finally, the GTP-dependent glutamate ligase CofE adds a variable-length γ-linked glutamate tail to produce mature F_420_ (22, 23). With the exception of the putative lactate kinase CofB, the enzymes responsible for F_420_ biosynthesis in archaea have been identified and characterized to various extents (1). Crystal structures have been obtained for CofC, CofD, and CofE from methanogenic archaea, providing some insight into how these enzymes function, but questions surrounding their catalytic mechanisms remain unresolved (23–25). For example, the crystal structure of CofD from Methanosarcina mazei was solved in the presence of Fo and GDP; however, no divalent cation(s) required for catalysis was present in the structure, and the ribosyl tail group of Fo, which receives the 2PL moiety from LPPG was disordered, precluding an understanding of the catalytic mechanism of this step in F_420_ biosynthesis (21, 25).

It was assumed that the biosynthesis pathway for archaeal F_420_ was generic to all F_420_-producing organisms (1). However, recent studies have shown that the structure and biosynthesis of F_420_ vary among F_420_-producing organisms (24, 26). F_420_ produced by the proteobacterial fungal symbiont Paraburkholderia rhizoxinica was found to incorporate 3-phospho-d-glycerate (3PG) in the place of 2PL, producing a chemically distinct F_420_ (26). In parallel, analysis of purified F_420_ biosynthesis enzymes from mycobacteria indicated that the central glycolytic and gluconeogenic intermediate phosphoenolpyruvate (PEP), rather than 2PL, is a precursor for F_420_ biosynthesis (24). In contrast to P. rhizoxinica, in mycobacteria, mature F_420_ is chemically analogous to that produced by archaea (27). All mycobacterial species possess the four enzymes required for F_420_ biosynthesis. However, as these enzymes catalyze reactions distinct from their archaeal homologues, the following alternative nomenclature is applied compared to the archaeal enzymes: FbiD (homologous to CofC), FbiC (a single protein with domains homologous to CofG and CofH), FbiA (homologous to CofD), and FbiB (N-terminal domain homologous to CofE) (8). In addition to its CofE-like domain, FbiB possesses an FMN-binding C-terminal domain, and biochemical evidence suggests that it is responsible for the reduction of the moiety derived from PEP (24, 28).

However, several findings have cast doubt on whether the proposed revised biosynthesis pathway of F_420_ is physiologically relevant. The predicted use of PEP in F_420_ biosynthesis in mycobacteria would lead to the formation of the oxidized intermediate compound dehydro-F_420_-0 (DH-F_420_-0). The production of DH-F_420_-0 was detected in a coupled enzyme assay containing purified FbiD and FbiA with PEP supplied as the substrate but has yet to be detected in mycobacterial cells (24). The study also showed that CofC from Methanocaldococcus jannaschii utilized PEP rather than 2PL for F_420_ biosynthesis (24), leading the authors to conclude that PEP is the general precursor for F_420_ biosynthesis in microorganisms. However, these findings contradict previous analysis of CofC activity in M. jannaschii cell lysates (19, 21), as well as recent biochemical analysis, which shows that CofC preferentially utilizes 2PL for F_420_ biosynthesis (26). In turn, these findings cast doubt on whether PEP is truly the preferred substrate for mycobacterial F_420_ biosynthesis and whether DH-F_420_-0 is the physiological intermediate in this pathway.

In this work, we first resolved this ambiguity by analyzing the F_420_ biosynthetic pathway in Mycobacterium smegmatis in whole cells. We demonstrate that PEP, not 2PL, is the substrate for F_420_ biosynthesis in mycobacterial cells, suggesting that divergent biosynthesis pathways are utilized to generate F_420_ in different microbial species. Consistent with this result, we determine that DH-F_420_-0 is the physiological intermediate for F_420_ biosynthesis in mycobacteria and that it is present in high quantities in cells lacking FbiB and comes bound to FbiA purified from M. smegmatis. Furthermore, to elucidate the catalytic mechanism for the formation of the novel intermediate DH-F_420_-0, we determined the crystal structure of FbiA in the presence and absence of its substrate and product compounds. These data resolve long-standing questions about the catalytic mechanism of FbiA and CofD in F_420_ biosynthesis. Moreover, they provide a target for therapeutic intervention through the inhibition of F_420_ biosynthesis, as well as insight into potential mechanisms for the emergence of delamanid and pretomanid drug resistance through mutations in FbiA.

## RESULTS

### Phosphoenolpyruvate is the substrate for the biosynthesis of F_420_ in mycobacterial cells.

**FIG 1:**
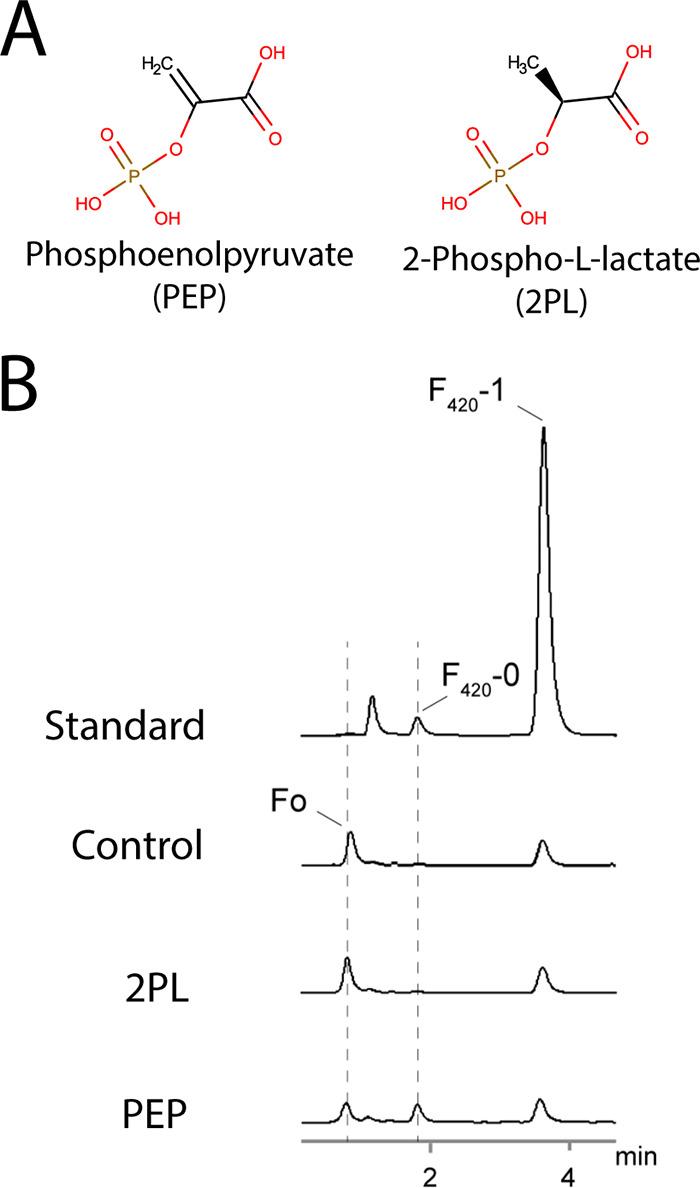
PEP, but not 2PL, stimulates DH-F_420_-0 synthesis in M. smegmatis cell lysates. (A) Two-dimensional (2D) structures of PEP and 2PL demonstrating the difference (double bond or single bond) in bonding between carbon 2 and 3. (B) Fluorescence emission detection chromatogram from HPLC of M. smegmatis lysates spiked with either 2PL or PEP or an unspiked control. Synthesis of a species with characteristic F_420_ fluorescence (excitation, 420 nm; emission, 480 nm) corresponding to F_420_-0 from the purified standard was detected only in the PEP-spiked lysate. The appearance of this F_420_-0-like species coincided with a decrease in the presence of Fo, suggesting that PEP is the precursor for F_420_ synthesis in M. smegmatis in cells. F_420_-1 in the standard corresponds to F_420_ with a single glutamate moiety.

To determine whether PEP or 2PL is the substrate for F_420_ biosynthesis in mycobacteria (Fig. 1A), we spiked clarified cell lysates from M. smegmatis with GTP and either PEP or 2PL, and monitored the synthesis of new F_420_ species through fast-performance liquid chromatography (FPLC) coupled with fluorescence detection. In cell lysates spiked with PEP, a species corresponding to F_420_-0 in the F_420_ standard was present, which was absent from both the untreated and 2PL-spiked lysates (Fig. 1B). The formation of this F_420_-0-like species in PEP-spiked lysates corresponded to a decrease in Fo levels, suggesting that synthesis of DH-F_420_-0 from PEP is occurring (Fig. 1B). These data strongly suggest that PEP, not 2PL, is the precursor for F_420_ biosynthesis in M. smegmatis.

**FIG 2:**
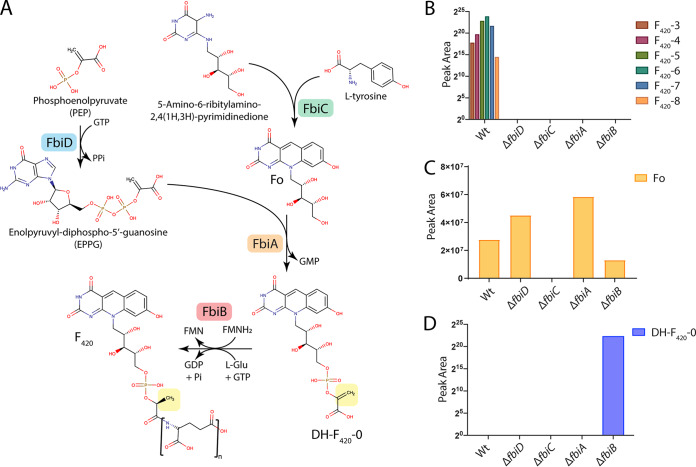
Mutagenic dissection of the F_420_ biosynthesis pathway in M. smegmatis reveals that DH-F_420_-0 is the biosynthetic intermediate in mycobacteria. (A) A schematic of the F_420_ biosynthesis pathway in M. smegmatis with PEP, rather than 2PL, utilized by FbiD to create the reaction intermediate EPPG. The enzymes responsible for catalytic steps are shown, along with the 2D structures of proposed pathway intermediates and mature F_420_. The yellow box highlights the reduction of DH-F_420_-0, proposed to be mediated by the C-terminal domain of FbiB using FMNH_2_. (B to D) LC-MS detection of mature F_420_ species (B), Fo (C), and DH-F_420_-0 (D) in M. smegmatis cell lysates of the wild type (Wt) and F_420_ biosynthesis pathway mutants confirming the proposed function of the F_420_ biosynthetic genes detecting the novel intermediate DH-F_420_-0 in whole cells. F_420_-*X* species in panel B correspond to different lengths of the polyglutamate chain where *X* = *n* tail length.

While the lysate spiking experiment establishes that PEP is specifically utilized for F_420_ synthesis in M. smegmatis, the fluorescence detection method utilized does not chemically differentiate between F_420_-0 or DH-F_420_-0. As PEP is utilized, it would be expected that DH-F_420_-0 is produced. However, DH-F_420_-0 may be rapidly reduced to F_420_-0 rather than accumulating in the cell. To confirm the synthesis of the DH-F_420_-0 in M. smegmatis, we created isogenic deletions in the four F_420_ biosynthesis genes: *fbiD*, *fbiC*, *fbiA*, and *fbiB* (Fig. 2A). The genome sequences of these deletion strains were determined, confirming clean deletion with no secondary mutations present. We then detected the deazaflavin species present in clarified cell lysates from these strains using fluorescence-coupled high-performance liquid chromatography (HPLC) and liquid chromatography coupled to mass spectrometry (LC-MS). As expected, based on the proposed function of these enzymes, mature F_420_ was detected only in wild-type cell lysates and possessed a polyglutamate tail length of three to eight (Fig. 2B; see also Fig. S1 in the supplemental material). Fo was detected in the wild type and all mutants except the *ΔfbiC* mutant, consistent with the function of this enzyme in the synthesis of the Fo-deazaflavin moiety (Fig. 2A and C and Fig. S1). The proposed biosynthetic intermediate DH-F_420_-0 was detected only in cell lysates of the *ΔfbiB* strain (Fig. 2D and Fig. S1). No F_420_-0 was detected in wild-type or mutant strains.

10.1128/mSystems.00389-20.1 FIG S1 Fluorescence-coupled HPLC analysis of clarified cell lysates from M. smegmatis F_420_ biosynthesis pathway mutants. Fluorescence (excitation wavelength [Ex λ] of 420 nm; emission wavelength [Em λ] of 480 nm) trace for wild-type (A), *ΔfbiC* (B), *ΔfbiA* (C), *ΔfbiD* (D), and *ΔfbiB* (E) strains showing the formation of mature F_420_ species in the wild-type strain only and accumulation of DH-F_420_-0 in the *ΔfbiB* strain. LU, fluorescence intensity. For *ΔfbiC*, shown in panel B, mass spectrometry analysis confirmed that no Fo or F_420_ species were present, despite observed low-level fluorescence. Download FIG S1, TIF file, 1.4 MB. Copyright © 2020 Grinter et al. 2020 Grinter et al. This content is distributed under the terms of the Creative Commons Attribution 4.0 International license.

The presence of DH-F_420_-0 (and absence of detected F_420_-0) in whole cells demonstrates that it is the central physiological intermediate in mycobacterial F_420_ biosynthesis. This also lends support to the biochemical and cellular assays indicating that PEP, not 2PL, is the substrate for this pathway in mycobacteria. Furthermore, in addition to its role as the F_420_ glutamyl-ligase, structural and biochemical analysis suggests that FbiB is responsible for the reduction of DH-F_420_-0 (24, 28). The detection of DH-F_420_-0 only in the *ΔfbiB* strain demonstrates that this intermediate is rapidly turned over in the cell and supports the hypothesis that FbiB and not another enzyme performs this step in mycobacterial F_420_ biosynthesis.

### FbiA copurifies with its product dehydro-F_420_-0.

In order to determine the catalytic mechanism for the synthesis of the novel intermediate DH-F_420_-0, we overexpressed and purified FbiA from M. smegmatis. Purified FbiA from M. smegmatis possessed a light yellow color, indicating copurification with a product or substrate molecule (Fig. S2A). The nature of this substrate was investigated using fluorescence spectroscopy, with purified FbiA found to have a broad absorbance peak at 400 nm and a corresponding emission peak at 470 nm (Fig. S2B), which is consistent with the presence of a deazaflavin with a protonated 8-OH group (16). We then utilized LC-MS to identify the deazaflavin species associated with FbiA and found that the major species was its product DH-F_420_-0 (Fig. S2C). In addition, significant quantities of mature F_420_ species were also associated with FbiA, suggesting that it also binds to mature F_420_ present in the cytoplasm (Fig. S2C).

10.1128/mSystems.00389-20.2 FIG S2 FbiA recombinantly expressed in M. smegmatis copurifies with its product DH-F_420_-0. (A) Purified, concentrated (16 mg · ml^−1^) recombinant FbiA produced in M. smegmatis showing a characteristic yellow color associated with a bound F_420_ species. (B) The absorbance (purple) and fluorescence spectra (blue) of purified FbiA from panel A, which is characteristic of F_420_ species with a protonated deazaflavin 8-OH group. (C) LC-MS analysis of F_420_ species bound to purified FbiA, showing that DH-F_420_-0 is the predominantly bound species and the presence of some mature F_420_ species. (D) SEC-MALS analysis of purified FbiA shows that it has a molecular weight in solution consistent with a homodimeric species. Download FIG S2, TIF file, 2.3 MB. Copyright © 2020 Grinter et al. 2020 Grinter et al. This content is distributed under the terms of the Creative Commons Attribution 4.0 International license.

### The crystal structure of FbiA reveals an active site with open and closed states.

**FIG 3:**
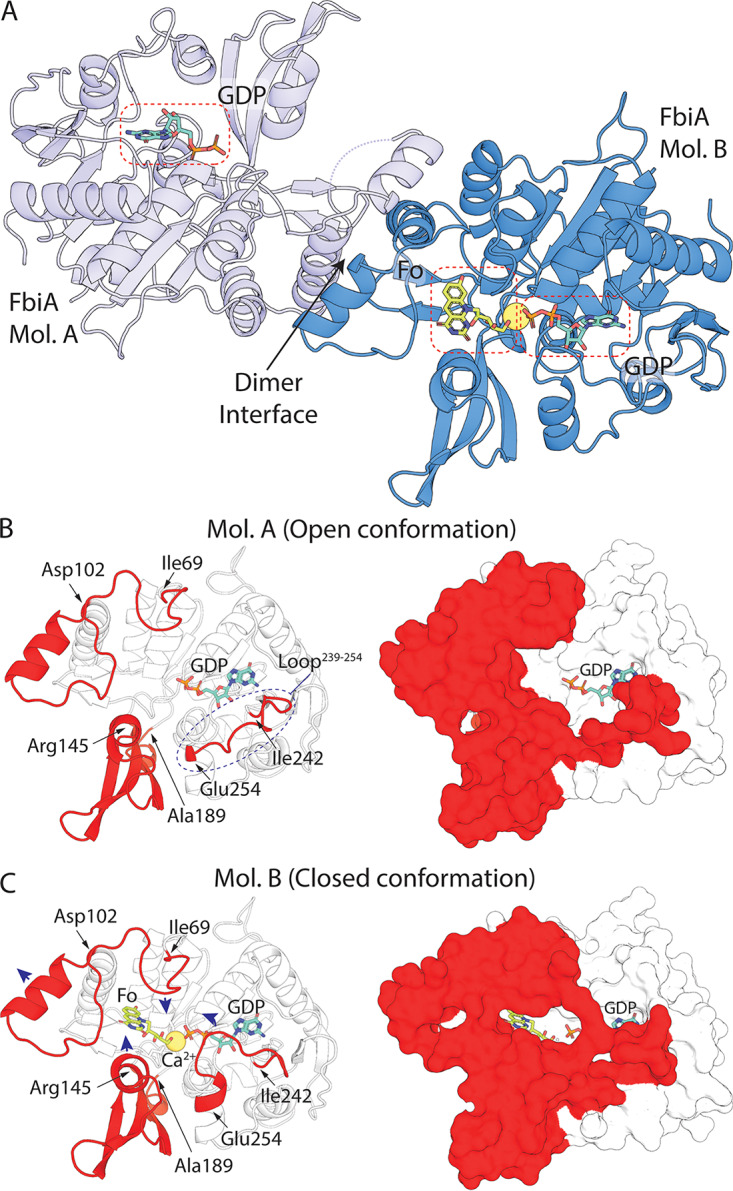
The crystal structure of FbiA captures the enzyme in open and closed states. (A) The crystal structure of FbiA from M. smegmatis in complex with Fo and GDP. FbiA is shown as a cartoon representation with molecule B (Mol. B) in sky blue and Mol. A in light blue. GDP and Fo are shown as stick representations, and Ca^2+^ is shown as a yellow sphere. (B) Mol. A from the FbiA structure exists in an open conformation. (Left) Mol. A as a cartoon with loops and subdomains which differ in conformation in Mol. B highlighted in red. (Right) Mol. A as a surface representation with mobile regions highlighted in red. (C) Mol. B of FbiA structure exists in a closed “catalytically ready” state. (Left) Mol. B displayed as in panel B, with the direction of movement of loops compared to Mol. A shown with blue arrows. (Right) Mol. B as in panel B, demonstrating how the mobile regions enclose the FbiA active site.

In order to resolve the catalytic mechanism of DH-F_420_-0 synthesis, purified FbiA was crystallized, and its structure was determined at 2.3 Å by X-ray crystallography (see Table S1 in the supplemental material). FbiA crystallized as a dimer mediated by the interaction of three α-helices and a β-sheet (Fig. 3A). This dimer is predicted to be stable by the protein-interaction prediction program PISA (29), and the molecular weight of FbiA determined by size exclusion chromatography coupled to multiangle light scattering (SEC-MALS) shows that it forms a dimer in solution (Table S2 and Fig. S2D). The structure of CofD from M. mazei, a homologous enzyme that instead utilizes LPPG derived from 2PL as its substrate, also crystallized as a dimer with an analogous interface to FbiA (25). Despite the copurification of FbiA with DH-F_420_-0, only weak electron density attributable to DH-F_420_-0 was observed in the catalytic site of molecule B (Mol. B) of the FbiA dimer (Fig. S3). To obtain the product-bound structure of FbiA, DH-F_420_-0 was purified from recombinant FbiA and soaked into existing crystals of FbiA. Using this procedure, electron density clearly attributable to the Fo and phosphate moieties of DH-F_420_-0 was observed in Mol. B of FbiA, allowing modeling of the product-bound structure (Fig. S3). Density for the carbonyl group of DH-F_420_-0 was less well resolved, suggesting that it exists in multiple conformations in product-bound FbiA (Fig. S3). Similarly, FbiA crystals were soaked with Fo and GDP, individually or in combination, and structures of substrate-bound FbiA were determined (Fig. S3 and Table S1).

10.1128/mSystems.00389-20.3 FIG S3 Electron density corresponding to FbiA substrates and products in cocrystal structures. The panel corresponds to the cocrystal structures indicated in the bottom right. A composite omit map is shown carved to visible molecules at a distance of 2 Å and contoured to 1 σ. Download FIG S3, TIF file, 1.8 MB. Copyright © 2020 Grinter et al. 2020 Grinter et al. This content is distributed under the terms of the Creative Commons Attribution 4.0 International license.

10.1128/mSystems.00389-20.6 TABLE S1 Crystallographic data collections and refinement statistics. Download Table S1, XLSX file, 0.01 MB. Copyright © 2020 Grinter et al. 2020 Grinter et al. This content is distributed under the terms of the Creative Commons Attribution 4.0 International license.

10.1128/mSystems.00389-20.7 TABLE S2 FbiA dimer interface statistics reported by PISA (29). Download Table S2, XLSX file, 0.01 MB. Copyright © 2020 Grinter et al. 2020 Grinter et al. This content is distributed under the terms of the Creative Commons Attribution 4.0 International license.

Comparison of the Mol. A and Mol. B from the FbiA dimer reveals the active site of the enzyme in distinct open and closed conformations (Fig. 3). In Mol. A, the active site is locked in an open state due to participation of an extended loop (amino acids [aa] 239 to 254) in crystal packing (Fig. 3B). In this open state, FbiA has a lower apparent substrate affinity, with no density attributable to Fo or DH-F_420_-0 and only weak density for GDP observed in the respective cocrystal structures (Fig. S3). In contrast, in Mol. B, the extended loop (aa 239 to 254) is partially disordered in the non-GDP-bound structures and encloses GDP in the active site in GDP-bound structures (Fig. 3C). In Mol. B, additional conformational changes are observed in amino acids 69 to 102 and a subdomain composed of amino acids 145 to 189, creating the binding pocket for the deazaflavin moiety of Fo or DH-F_420_-0, which is not present in Mol. A (Fig. 3B and C). The conformational differences observed between Mol. A and Mol. B are consistent in the apo-, substrate-, and product-bound structures, demonstrating that they are not substrate induced and are likely representative of conformational differences of the enzyme in solution.

### The crystal structures of FbiA in substrate- and product-bound forms provide mechanistic insight into dehydro-F_420_-0 synthesis.

**FIG 4:**
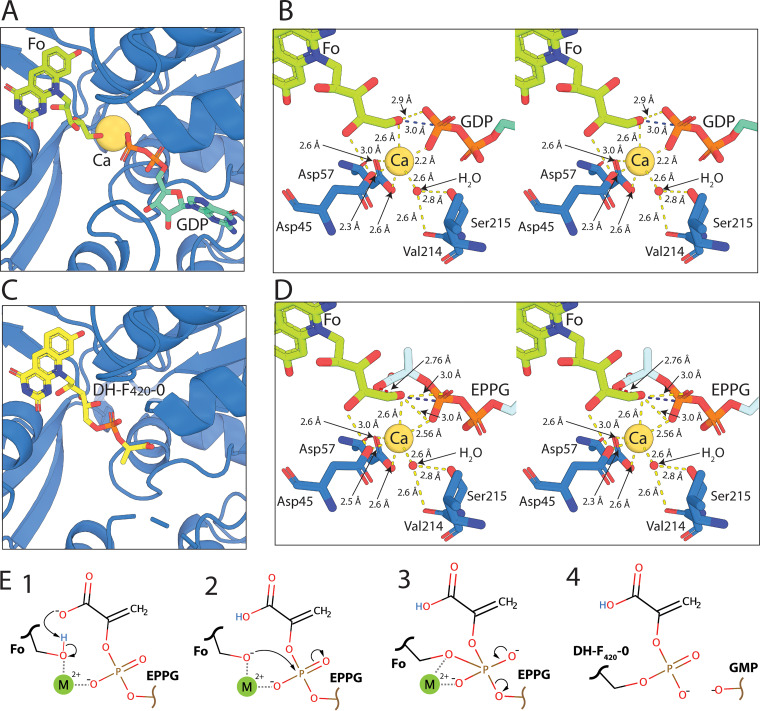
Resolution of the structure of FbiA in the presence of Fo, GDP, and DH-F_420_-0 provides insight into its catalytic mechanism. (A) Fo and GDP in complex with Mol. B of FbiA in coordination with the catalytic Ca^2+^ ion. FbiA is shown as a sky blue cartoon, Fo and GDP as sticks, and Ca^2+^ as a sphere. (B) Stereoview of the catalytic center of the FbiA active site in complex with Fo and GDP, showing FbiA side chains involved in coordinating the catalytic metal ion and a coordinating H_2_O molecule. Bond distances of <3.2 Å are shown as yellow dashed lines, and the distance between the terminal OH of Fo and P of the β-phosphate of GDP is highlighted in blue. (C) DH-F_420_-0 in complex with FbiA, shown as in panel A. (D) Stereoview of the FbiA catalytic center with the reaction substrate EPPG model in place of GDP displayed in panel C, with the close proximity between the carboxylic acid group of EPPG and the terminal OH of Fo highlighted with a red dashed line. (E) Schematic showing the proposed catalytic mechanism for the formation of DH-F_420_-0 by FbiA.

The resolution of the structure of FbiA in the presence of its substrate and product compounds provides key insights into the catalytic mechanism of this unique phosphotransferase. It was previously established that FbiA and its archaeal homologue CofD require the presence of the divalent cation Mg^2+^ for activity (21, 24). However, it remained to be resolved whether Mg^2+^ is bound stably in the FbiA active site during catalysis and the mode of coordination of the ion(s). In the GDP-bound structures of FbiA, a single metal ion was present in the active site of Mol. B of FbiA (Fig. 4A; see also Fig. S4A and Fig. S5). Interestingly, no metal ion was observed in Mol. A, despite the presence of GDP, suggesting that its recruitment is conformation dependent (Fig. S2). As calcium acetate is present at high concentration (0.2 M) in the crystallization condition, and magnesium is absent, we modeled this ion as Ca^2+^. The activity of FbiA in the presence of Ca^2+^ has not been tested. Ca^2+^ and Mg^2+^ have been shown to be interchangeable in some phosphohydrolase enzymes, while for some Mg^2+^-dependent kinases, Ca^2+^ is a competitive inhibitor of activity (30, 31). Despite this, the coordination of Ca^2+^ is very similar to Mg^2+^, allowing for analysis of the enzyme active site with Ca^2+^ bound (31). In the GDP-only structure of FbiA, the Ca^2+^ ion is directly coordinated by aspartates 45 and 57, an oxygen atom of the β-phosphate of GDP, two H_2_O molecules, and a glycerol molecule (Fig. S4B). Aspartate 57 exhibits bidentate coordination of the Ca^2+^ ion, leading to a coordination number of seven with distorted octahedral geometry.

10.1128/mSystems.00389-20.4 FIG S4 The crystal structure of FbiA in complex with Fo and GDP. (A) GDP in complex with Mol. B of FbiA. FbiA is represented as a sky blue cartoon, bound GDP and glycerol molecules are shown as stick models, and bound Ca^2+^ ion is shown as a yellow sphere. (B) A stereoview of the catalytic center of the FbiA active site in complex with GDP and glycerol, showing FbiA side chains involved in coordinating the catalytic metal ion and a coordinating H_2_O molecule. Bond distances of <3.2 Å are shown as yellow dashed lines. (C) Fo in complex with Mol. B of FbiA is shown as a sky blue cartoon, and Fo is shown as a stick model. Download FIG S4, TIF file, 2.7 MB. Copyright © 2020 Grinter et al. 2020 Grinter et al. This content is distributed under the terms of the Creative Commons Attribution 4.0 International license.

10.1128/mSystems.00389-20.5 FIG S5 The full interaction network of the FbiA active site in the presence of substrates (Fo and GDP) and product (DH-F_420_-0). Download FIG S5, TIF file, 2.0 MB. Copyright © 2020 Grinter et al. 2020 Grinter et al. This content is distributed under the terms of the Creative Commons Attribution 4.0 International license.

In the GDP and Fo-bound structure, the coordination of Ca^2+^ is analogous to the GDP-only structure. However, the glycerol molecule and one of the H_2_O molecules observed in the GDP-only structure are displaced by the ribosyl chain of Fo, resulting in a coordination number of six with octahedral geometry (Fig. 4B and Fig. S4B). The terminal hydroxyl group of Fo is significantly closer to the Ca^2+^ ion (2.6 Å) and to the β-phosphate of GDP (2.8 Å from O and 3.0 Å from P) than the coordinating hydroxyl of glycerol, which is not within the bonding distance of GDP. These bond distances between the hydroxyl of Fo and GDP, as well as the central orientation of the hydroxyl of Fo toward the β-phosphate of GDP, place it in an ideal position to act as the acceptor substrate for the transfer of PEP catalyzed by FbiA (Fig. 4B). In the Fo- and DH-F_420_-0-bound structures, no density corresponding to a Ca^2+^ ion was observed (Fig. 4C and Fig. S4C). This suggests that binding of FbiA to its catalytic metal ion is contingent on complex formation with enolpyruvyl-diphospho-5ʹ-guanosine (EPPG) (Fig. 2A), which is substituted for GDP in our structures due to the instability of the F_420_ pathway intermediate (24). The ability of FbiA to bind Fo in the absence of GDP and Ca^2+^ suggests that substrate binding to FbiA is not sequential; however, recruitment of all three components is required for catalysis to proceed.

On the basis of these structural data and previous biochemical characterization of FbiA and CofD, we propose a catalytic mechanism for synthesis of the DH-F_420_-0 (Fig. 4E) (21, 25). Our structural data agree with previous work that suggests that CofD does not form a covalent intermediate as part of the reaction mechanism (21), but rather the reaction proceeds through direct nucleophilic attack of the β-phosphate of EPPG by the terminal hydroxyl of Fo. This leads to the formation of a pentavalent transition state between Fo and EPPG that is stabilized by the catalytic metal ion (Fig. 4E). In order for the hydroxyl group of Fo to perform nucleophilic attack, it needs to be activated through deprotonation. The carboxylic acid group of EPPG is a likely candidate for this activation, as it is the only acidic group in close proximity to the hydroxyl group of Fo when EPPG is modeled in the FbiA structure in place of GDP (Fig. 4D). Additionally, the activation of Fo by the carboxylic acid group of EPPG would provide FbiA with substrate specificity for EPPG over GDP and GTP. Following the formation of the pentavalent reaction intermediate, GMP would act as the leaving group, leading to the formation of the phosphodiester bond between PEP and Fo and the formation of DH-F_420_-0 (Fig. 4E).

## DISCUSSION

Integrating these findings with other recent literature, it is now clear that the substrate for the initial stage of F_420_ tail biosynthesis differs between F_420_-producing organisms (19, 24, 26). We definitively show here that, in mycobacteria, PEP is the substrate for F_420_ biosynthesis, resolving the ambiguity in the literature (24, 26). In contrast, in the archaeal and proteobacterial species that have been analyzed, 2PL and 3PG, respectively, are preferentially utilized (19, 26). This divergent substrate utilization occurs despite the enzymes responsible for this stage of synthesis (FbiD/CofC and FbiA/CofD) sharing a common evolutionary history (8). This suggests that the substrate specificity of these enzymes has evolved in response to selection to maintain compatibility between the substrate used for F_420_ biosynthesis and what is available in the cellular metabolite pool. Both PEP and 3PG are intermediates in central metabolic pathways, including glycolysis, whereas 2PL is not thought to be present in significant quantities in most organisms (24, 26, 32). This makes PEP and 3PG compatible substrates for F_420_ biosynthesis in *Mycobacterium* spp. and P. rhizoxinica, respectively, with the specifics of cellular metabolism of each organism likely dictating which compound was selected for F_420_ biosynthesis. In contrast, in the archaeon Methanobacterium thermoautotrophicum, 2PL is present at micromolar concentrations (19). However, in archaeal species, it remains to be determined how 2PL is synthesized and whether this compound plays a wider role as a general metabolite beyond F_420_ biosynthesis.

Phylogenetic analysis of FbiD/CofC and FbiA/CofD suggests that these proteins were horizontally transferred between bacteria and archaea (8). Based on this analysis, it is curious that mycobacteria reduce DH-F_420_-0 produced via PEP to F_420_, rendering it chemically identical to that produced with 2PL. The redox properties of the deazaflavin group of DH-F_420_ and F_420_ are identical, and chemically the molecules are very similar, posing the question: why is reduction of DH-F_420_-0 is required? A plausible explanation is that actinobacteria originally utilized 2PL for F_420_ synthesis, with a switch to PEP occurring at a later stage in evolution. As a result, the F_420_-dependent enzymes present in mycobacteria evolved to recognize the nonplanar 2PL moiety of F_420_, requiring reduction of DH-F_420_ to maintain compatibility after the substrate switch. Previous structural and biochemical analysis suggests that the C-terminal domain of mycobacterial FbiB is responsible for the reduction of DH-F_420_-0 (24, 28). This domain is present in all mycobacterial species but is absent from FbiB/CofE in most other F_420_-producing organisms, including *M. mazei* and *P. rhizoxinica*, that produce F_420_ through pathways that do not require this reductive step (8, 26). This conclusion is supported by our cellular analysis of F_420_ biosynthesis in M. smegmatis, which shows that DH-F_420_-0 accumulates in the *ΔfbiB* strain (Fig. 2A and D).

The structural analysis of FbiA that we present in this work provides unprecedented insight into the catalytic mechanism for the novel phosphotransferase reaction employed at this step in F_420_ biosynthesis. The crystal structure of FbiA shows that this enzyme employs a flexible active site to capture Fo and EPPG, precisely positioning them for catalysis. Determination of the fully resolved FbiA substrate complex, in the presence of a single catalytic metal ion, provides a clear picture of the mechanism of catalysis of this enzyme. In this structure, the terminal hydroxyl group of Fo is ideally positioned for nucleophilic attack of the β-phosphate of EPPG, strongly suggesting that DH-F_420_-0 biosynthesis occurs through direct transfer of PEP to Fo, via a pentavalent phosphate intermediate that is stabilized by the catalytic metal ion. The positioning of the Fo terminal hydroxyl group in our structure in relation to EPPG is strikingly similar to that of the attacking ribose in the final step in DNA ligation by T4 ligase, recently resolved by X-ray crystallography (33). This is consistent with both reactions resulting in the formation of a phosphodiester bond through direct nucleophilic attack of a diphosphonucleoside intermediate. As no acidic side chains are present in proximity of the terminal hydroxyl of Fo in our structure of FbiA, it is likely that deprotonation of this group for nucleophilic attack is EPPG induced, possibly by the PEP carboxyl moiety. These data are also consistent with biochemical analysis of CofD from *M. mazei*, which did not detect the formation of a catalytic reaction intermediate during the synthesis of F_420_-0 (21).

The resolution of the F_420_ biosynthesis pathway also has implications for tuberculosis treatment. It has been proposed that F_420_ biosynthesis represents a promising target for the development of drugs for the treatment of M. tuberculosis, given the pleiotropic role of this cofactor and its absence from human cells. While nonessential for the growth of mycobacteria under optimal conditions, F_420_ has been shown to be important for persistence, recovery from dormancy, and antibiotic resistance, and hence is likely to contribute to M. tuberculosis infection (1). Given that FbiA mediates the key step in F_420_ biosynthesis, the structural insights into FbiA catalysis provide a basis for the development of inhibitory compounds targeting F_420_ biosynthesis (24). In addition, loss of function of FbiA causes resistance to the clinical nitroimidazole prodrugs delamanid and pretomanid, both of which are activated by the F_420_H_2_-dependent reductase Ddn (15, 17, 34). Hence, the structural and mechanistic insights provided here will enable prediction of which substitutions are likely to impair or inactivate FbiA, thus conferring resistance to these compounds.

## MATERIALS AND METHODS

### Creation of M. smegmatis F_420_ biosynthesis mutant strains.

M. smegmatis MSMEG_5126 was deleted in wild-type M. smegmatis mc^2^155 using a two-step allelic replacement strategy. Two 0.8-kb fragments containing sequences from the left and right flanking regions of the MSMEG_5126 (*fbiC*) gene were cloned as separate constructs and later combined to make the deletion construct. The left flanking fragments were amplified using ProofStart DNA polymerase (Qiagen) with primers MSMEG_5126left and MSMEG_5126leftrev, and the PCR product was subsequently cloned into the SacI/BamHI sites of pUC18, creating plasmid pUC-MSMEG_5126left (additional information on the primers in Table S3 in the supplemental material). A 0.8-kb fragment containing sequence from the right side of MSMEG_5126 was amplified using primers MSMEG_5126right and MSMEG_5126rightrev and cloned into the XbaI/BamHI sites of pUC18, creating plasmid pUC-MSMEG_5126right. The right flanking sequence was then excised from pUC-MSMEG_5126right using XbaI/SacI and subcloned into Xba/SacI-digested pUC-MSMEG_5126left, fusing the left and right flanking sequences to create plasmid pUC-ΔMSMEG_5126.

10.1128/mSystems.00389-20.8 TABLE S3 Oligonucleotide primers utilized in this study. Download Table S3, XLSX file, 0.01 MB. Copyright © 2020 Grinter et al. 2020 Grinter et al. This content is distributed under the terms of the Creative Commons Attribution 4.0 International license.

The 1.6-kb fused insert was then liberated using Xba/SacI and subcloned into Xba/SacI-digested pMSS vector (35), a suicide plasmid for M. smegmatis that contains streptomycin selection and sucrose counterselection markers. The resultant plasmid, pMSS:ΔMSMEG_5126, was sequenced and then electroporated into electrocompetent M. smegmatis mc^2^155 cells, using an ECM 630 electroporator (BTX), selecting for streptomycin-resistant colonies (30 μg/ml), which were then screened for sensitivity to 10% (wt/vol) sucrose. DNA from confirmed streptomycin-resistant, sucrose-sensitive colonies was PCR amplified using primer pair MSMEG_5126screen-F and MSMEG_5126KOright-R. The resultant PCR product was confirmed by DNA sequencing.

A confirmed single crossover (SCO) strain was grown for 3 days in the absence of antibiotic selection, serially diluted, and plated on LB plates containing 10% (wt/vol) sucrose to select for potential double crossover (DCO) strains (i.e., MSMEG_5126 deletion mutants). Genomic DNA was extracted from sucrose-resistant, streptomycin-sensitive clones, digested using ClaI/NcoI, and subjected to Southern blot analysis using an MSMEG_5126-specific probe to confirm deletion of the MSMEG_5126 gene. For Southern blotting, 2 μg of genomic DNA (gDNA) was digested with appropriate restriction enzymes (NEB) at 37°C for 16 h. Purified samples and digoxigenin (DIG)-labeled, HindIII-digested λ DNA markers were separated on a 1% agarose gel, followed by depurination, denaturation, neutralization, and capillary transfer onto a nylon membrane (Thermo Fisher). The membrane was then hybridized at 67°C with a gene-specific probe prepared by DIG labeling a 3.0-kb PCR product obtained using primers MSMEG_5126screen-R and MSMEG_5126screen-F. Once confirmed by Southern blotting, the genomes of mutant strains were sequenced at the Peter Doherty Institute for Infection and Immunity at the University of Melbourne, and mapped to the wild-type strain, confirming that the strains were otherwise isogenic.

MSMEG_1829 (*fbiB*), MSMEG_2392 (*fbiD*), and MSMEG_1830 (*fbiA*) deletion mutants were generated by using the same methods used for MSMEG_5126, using gene-specific primer combinations (Table S3). Individual deletion mutants of *fbiA*, *fbiB*, and *fbiD* were confirmed by Southern blotting following digestion with PvuII and then genome sequencing.

### Purification of Fo, DH-F_420_-0, and F_420_.

Fo was purified from culture supernatants of M. smegmatis mc^2^155 overexpressing FbiC from M. tuberculosis cloned into the acetamide-inducible vector pMyNT. The cells were grown at 37°C in 7H9 medium to an optical density at 600 nm (OD_600_) of ∼3.0 before FbiC expression was induced by the addition of 0.2% acetamide. The cells were grown for an additional 72 h at 37°C with shaking, and supernatant was clarified by centrifugation at 10,000 × *g* for 20 min. The clarified supernatant was filtered (0.45 μm) and applied to a C_18_-silica column equilibrated in distilled H_2_O (dH_2_O). Bound Fo was eluted with 20% methanol in dH_2_O, and the solvent was removed by vacuum evaporation. Fo was resuspended in dH_2_O and centrifuged (20,000 × *g* for 20 min) to remove insoluble contaminants, before reapplication to a C_18_-silica column equilibrated in dH_2_O. Fo was again eluted with 20% methanol in dH_2_O, vacuum evaporated, and stored at –20°C for further analysis.

F_420_ was expressed and purified as previously described in M. smegmatis mc^2^4517 overexpressing FbiA, FbiB, and FbiC in the expression vector pYUBDuet-FbiABC (36). The cells were grown in LB broth plus 0.05% Tween 80 at 37°C with shaking to an OD_600_ of ∼2.0 before the expression of the *fbi* genes was induced with 0.2% acetamide. The cells were grown for an additional 72 h before harvesting by centrifugation at 10,000 × *g* for 20 min. The cells were resuspended in 50 mM Tris (pH 7.5) at a ratio of 10 ml of buffer per 1 g of cells (wet weight) and lysed by autoclaving. The autoclaved cell suspension was clarified by centrifugation at 20,000 × *g* for 20 min. The clarified supernatant was applied to a High Q Anion Exchange Column (Bio-Rad) equilibrated in 50 mM Tris (pH 7.5). Bound species were eluted with a gradient of 0 to 100% of 50 mM Tris and 1 M NaCl (pH 7.5). Fractions containing F_420_ were identified via visible spectroscopy based on their distinctive absorbance peak at 420 nm. Fractions containing F_420_ were pooled and applied to a C_18_-silica column equilibrated in dH_2_O. F_420_ was eluted with 20% methanol in H_2_O, vacuum evaporated, and stored at –20°C for further analysis.

DH-F_420_-0 was extracted from purified FbiA expressed in M. smegmatis as described below. Purified concentrated FbiA (∼20 mg ml^−1^; prior to cleavage of the polyhistidine tag) was denatured in a buffer containing 50 mM Tris and 8 M urea (pH 7.0). This solution containing denatured FbiA and free DH-F_420_-0 was applied to a nickel-agarose column, with denatured FbiA binding to the column due to its hexahistidine (hexahis) tag and DH-F_420_-0 eluting in the flowthrough. The flowthrough containing DH-F_420_-0 was applied to a Superdex 30 10/300 column equilibrated in dH_2_O, and eluted fractions containing DH-F_420_-0 were identified based on their absorbance at 420 nM. DH-F_420_-0-containing fractions were pooled, vacuum evaporated, resuspended in 500 μl of dH_2_O, and reapplied to the Superdex 30 10/300 column equilibrated in 20% acetonitrile in dH_2_O. DH-F_420_-0-containing fractions were then pooled, vacuum evaporated, and stored at –20°C for further analysis.

### FbiA expression and purification.

The DNA coding sequence corresponding to FbiA from M. smegmatis was amplified by PCR using the primers in Table S3, resulting in a DNA fragment with 5′ NcoI and 3′ HindIII sites, respectively. This fragment was cloned into pMyNT by restriction enzyme cloning using the aforementioned sites, yielding pMyNTFbiAMS, which expresses FbiA with a tobacco etch virus (TEV)-cleavable N-terminal hexahis tag. This vector was cloned and propagated in Escherichia coli DH5α in LB medium/agar with the addition of 200 μg ml^−1^ hygromycin B. Sequence-confirmed pMyNTFbiAMS was transformed into M. smegmatis mc^2^155 via electroporation, with successful transformants selected for in LB plus 0.05% Tween 80 (LBT) agar in the presence of 50 μg ml^−1^ hygromycin B. Colonies from this transformation were used to inoculate 50 ml of LBT medium plus 50 μg ml^−1^ hygromycin B, which was grown with shaking at 37°C until stationary phase (2 or 3 days). This starter culture was used to inoculate 5 liters of terrific broth plus 0.05% Tween 80 (TBT), giving a 1:100 dilution of the starter culture. The cells were grown with shaking at 37°C for 24 h until approximately mid-log phase, and protein production was induced through the addition of 0.2% acetamide. The cells were grown with shaking at 37°C for an additional 72 h before they were harvested via centrifugation at 5,000 × *g* for 20 min. Harvested cells were either lysed immediately or stored frozen at –20°C.

The cells were resuspended in Ni binding buffer (20 mM HEPES, 300 mM NaCl, 20 mM imidazole; pH 7.5) at a ratio of approximately 5 ml of buffer per 1 g of cells (wet weight). Lysozyme (1 mg ml^−1^), DNase (0.5 mg ml^−1^), and complete protease inhibitor tablets (Roche) were added, and cells were lysed with a cell disruptor (Constant Systems). The cell lysate was stored on ice and clarified by centrifugation at 4°C at 30,000 × *g*. Clarified lysate was passed through a column containing Ni^2+^ agarose resin equilibrated in Ni binding buffer. The column was washed with Ni binding buffer, and protein was eluted with a gradient of Ni gradient buffer (20 mM HEPES, 300 mM NaCl, 500 mM imidazole; pH 7.5). Fractions containing FbiA were identified based on absorbance at 280 nm and their yellow color due to F_420_ precursor copurification and pooled. Pooled fractions were applied to a Superdex S200 26/600 size exclusion chromatography (SEC) column, equilibrated with SEC buffer (20 mM HEPES, 150 mM NaCl; pH 7.5), and fractions containing FbiA were identified as described above and pooled. The hexahis tag was cleaved from purified FbiA through the addition of 0.5 mg of hexahistidine-tagged TEV protease (expressed and purified as described in reference 37) per mg of FbiA, plus 1 mM dithiothreitol (DTT). Digestion was performed at room temperature for ∼6 h before the sample was passed through a Ni^2+^ agarose column to remove TEV and the cleaved hexahis tag. The resulting flowthrough from this column was collected, concentrated to ∼15 mg ml^−1^, and snap-frozen at –80°C. Purified FbiA was light yellow in color due to copurification with F_420_ precursors, with a yield of 5 to 10 mg per liter of culture. The molecular weight of purified FbiA was determined by size exclusion coupled to multiangle laser light scattering (SEC-MALS), using a Superdex S200 Increase 10/300 column equilibrated in 200 mM NaCl and 50 mM Tris (pH 7.9), coupled to fast performance liquid chromatography (FPLC) (Shimadzu) with MALS detection (Wyatt Technology).

### FbiA crystallization, ligand soaking, and structure solution.

Purified FbiA was screened for crystallization conditions using a sparse matrix approach, with approximately 600 individual conditions screened. Thin intergrown plate crystals of FbiA formed under a number of conditions, with a condition containing 0.1 M Tris (pH 8.0), 0.2 M Ca acetate and 20% polyethylene glycol 3350 (PEG 3350) chosen for optimization. Diffraction quality crystals were obtained by microseeding into 0.1 M Tris (pH 8.5), 0.2 M Ca acetate, and 16% PEG 3350 with or without 20% glycerol. Crystals grew as bunches of very thin plates and were slightly yellow in color. Crystals from conditions containing glycerol were looped and directly flash cooled to 100 K in liquid N_2_, providing “apo” crystals for data collection. Crystals from non-glycerol-containing wells were transferred into well solution with 20% glycerol and either Fo, GDP, and Fo and GDP or DH-F_420_-0. Crystals were incubated in this solution for 1 to 5 min before they were looped and flash cooled to 100 K in liquid N_2_.

Data were collected at the Australian Synchrotron, with crystals diffracting anisotropically to ∼2.2 to 3.0 Å, and processed using XDS and merged using Aimless from the CCP4 package (38, 39). The structure of FbiA was solved by molecular replacement using Phaser (40), with a search model derived from the structure of CofD from *M. mazei* (PDB identifier [ID] 3C3D) prepared based on the amino acid sequence for FbiA from M. smegmatis using sculptor from the Phenix package (40). Native and ligand-soaked structures of FbiA were built and refined using Coot and phenix.refine from the Phenix package (40, 41). Structural coordinates for Fo, DH-F_420_-0 and F_420_ were generated using the AceDrg program within the CCP4 suite (39, 42).

### LC-MS detection of F_420_ and precursors.

Wild-type and Fbi mutant M. smegmatis mc^2^155 strains were grown in 20 ml of LBT medium until stationary phase (2 or 3 days) and harvested by centrifugation at 5,000 × *g* for 20 min. The cells were resuspended in 2 ml of dH_2_O and lysed by boiling for 5 min before clarification by centrifugation at 25,000 × *g* for 10 min. The soluble fraction was then decanted for mass spectrometry analysis. Samples were analyzed by hydrophilic interaction liquid chromatography (HILIC) coupled to high-resolution mass spectrometry (LC-MS) according to a previously published method (43). In brief, the chromatography utilized a guard (20 by 2.1 mm) in series with an analytical column (150 by 4.6 mm) (both ZIC-pHILIC; Merck). The column temperature was maintained at 25°C with a gradient elution of 20 mM ammonium carbonate (solution A) and acetonitrile (solution B) (linear gradient time−percent solution B as follows: 0 min−80%, 15 min−50%, 18 min−5%, 21 min−5%, 24 min−80%, 32 min−80%) on a Dionex RSLC3000 ultrahigh performance liquid chromatograph (UHPLC) (Thermo). The flow rate was maintained at 300 μl min^−1^. Samples were kept at 4°C in the autosampler, and 10 μl was injected for analysis. The mass spectrometric acquisition was performed at 35,000 resolution on a Q-Exactive Orbitrap MS (Thermo) operating in rapid switching positive (4 kV) and negative (−3.5 kV) mode electrospray ionization (capillary temperature, 300°C; sheath gas, 50; auxiliary [Aux] gas, 20; sweep gas, 2; probe temperature, 120°C). The resulting LC-MS data were processed by integrating the area below the extracted ion chromatographic peaks using TraceFinder 4.1 (Thermo Scientific). All species were detected in negative mode as the singly deprotonated anion (Fo and DH-F_420_-0) or in the case of the F_420_-n species the double deprotonated dianion.

### 2-Phospholactate synthesis.

Synthesis of 2-phospholactate was achieved in high-yield via one-step catalytic hydrogenation of phosphoenolpyruvate as previously described (44). Briefly, 26 mg of phosphoenolpyruvate monosodium salt and 12 mg palladium on carbon (Pd/C) (10% wet concentration) were transferred into a 25 ml round bottom flask and dissolved in 5 ml water. After fixing the flask with a hydrogen bag, the solution was stirred for 5 h at room temperature and 1 atm pressure. The solution was filtered, dried by rotary evaporation, and stored at –20°C. Successful production of a racemic mix of 2-phospholactate was verified by mass spectrometry and ^1^H NMR.

### Stimulation of DH-F_420_-0 production in spiked M. smegmatis cell lysates and detection of F_420_ species by HPLC.

To detect F_420_ synthesis in spiked M. smegmatis lysates, 500-ml cultures were grown in LBT for 3 days at 37°C with shaking. The cells were harvested by centrifugation at 8,000 × *g* for 20 min at 4°C. The pellet was resuspended in 50 ml lysis buffer (50 mM morpholinepropanesulfonic acid [MOPS], 1 mM phenylmethylsulfonyl fluoride [PMSF], 1 mM DTT, 5 mM MgCl_2_, 2.5 mg · ml^−1^ lysozyme, 2.5 mg DNase I). An M-110P Microfluidizer (Fluigent) pressure-lysis maintained at 4°C was used to lyse the cells. The lysate was centrifuged at 10,000 × *g* at 4°C for 20 min. One-milliliter aliquots of lysate were spiked with either 1 mM phosphate buffer (pH 7.0) plus GTP and 2PL or GTP and PEP. These spiked samples, along with a “no spike” control, were incubated at 4 h at 37°C. To terminate the reaction, the aliquots were heated at 95°C for 20 min and then centrifuged at 16,000 × *g* for 10 min. The supernatants were filtered through a 0.22-μm polyvinylidene difluoride (PVDF) filter and moved to analytical vials.

F_420_ biosynthetic intermediates present in the filtered M. smegmatis cell lysates were analyzed by separation and detection using an Agilent 1200 series HPLC system equipped with a fluorescence detector and a Poroshell 120 EC-C_18_ column (2.1 by 50 mm; 2.7 μm). The system was run at a flow rate of 0.3 ml min^−1^, and the samples were excited at 420 nm, and emission was detected at 480 nm. A gradient of two buffers were used: buffer A containing 20 mM ammonium phosphate and 10 mM tetrabutylammonium phosphate, pH 7.0, and buffer B, 100% acetonitrile. A gradient was run from 25% to 40% buffer B as follows: 0 to 1 min, 25%; 1 to 10 min, 25% to 35%; 10 to 13 min, 35%; 13 to 16 min, 35 to 40%; and 16 to 19 min, 40% to 25%.

### Data availability.

The crystallographic coordinates and associated structure factors for the FbiA structures produced in this study are available in the Protein Data Bank (PDB) with the following accession codes: FbiA Apo, 6UVX; FbiA plus Fo, 6UW1; FbiA plus GDP, 6UW3; FbiA plus GDP plus Fo, 6UW5; FbiA plus DH-F420-0, 6UW7.
