# Supplementary figures and images for: Cellular and Structural Basis of Synthesis of the Unique Intermediate Dehydro-F420-0 in Mycobacteria
Source: mSystems. 2020 May 19;5(3):e00389-20. doi: 10.1128/mSystems.00389-20 (PMC7253369; doi:10.1128/mSystems.00389-20)

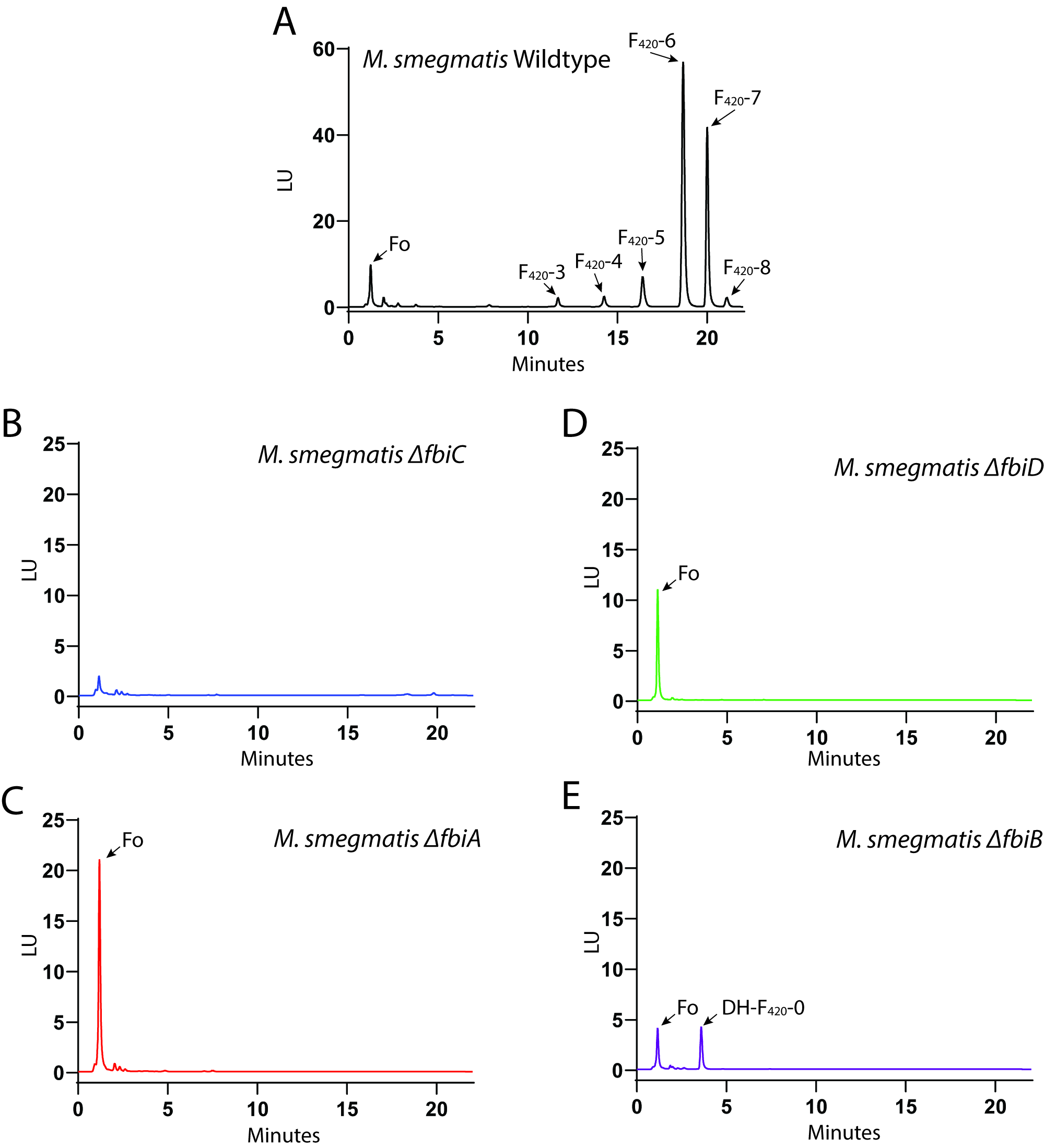

Supplement: FIG S1 [file mSystems.00389-20-sf001.tif]

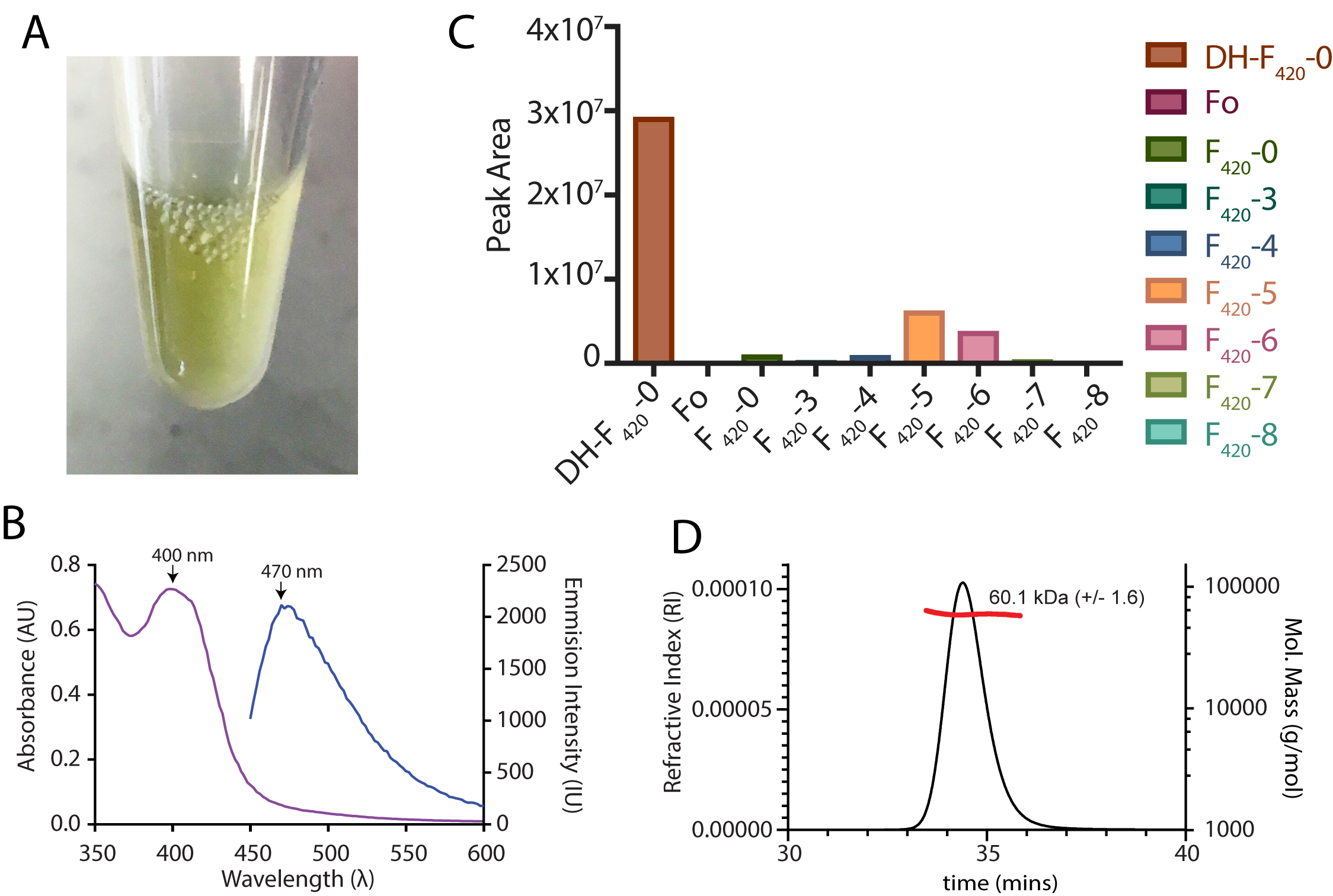

Supplement: FIG S2 [file mSystems.00389-20-sf002.tif]

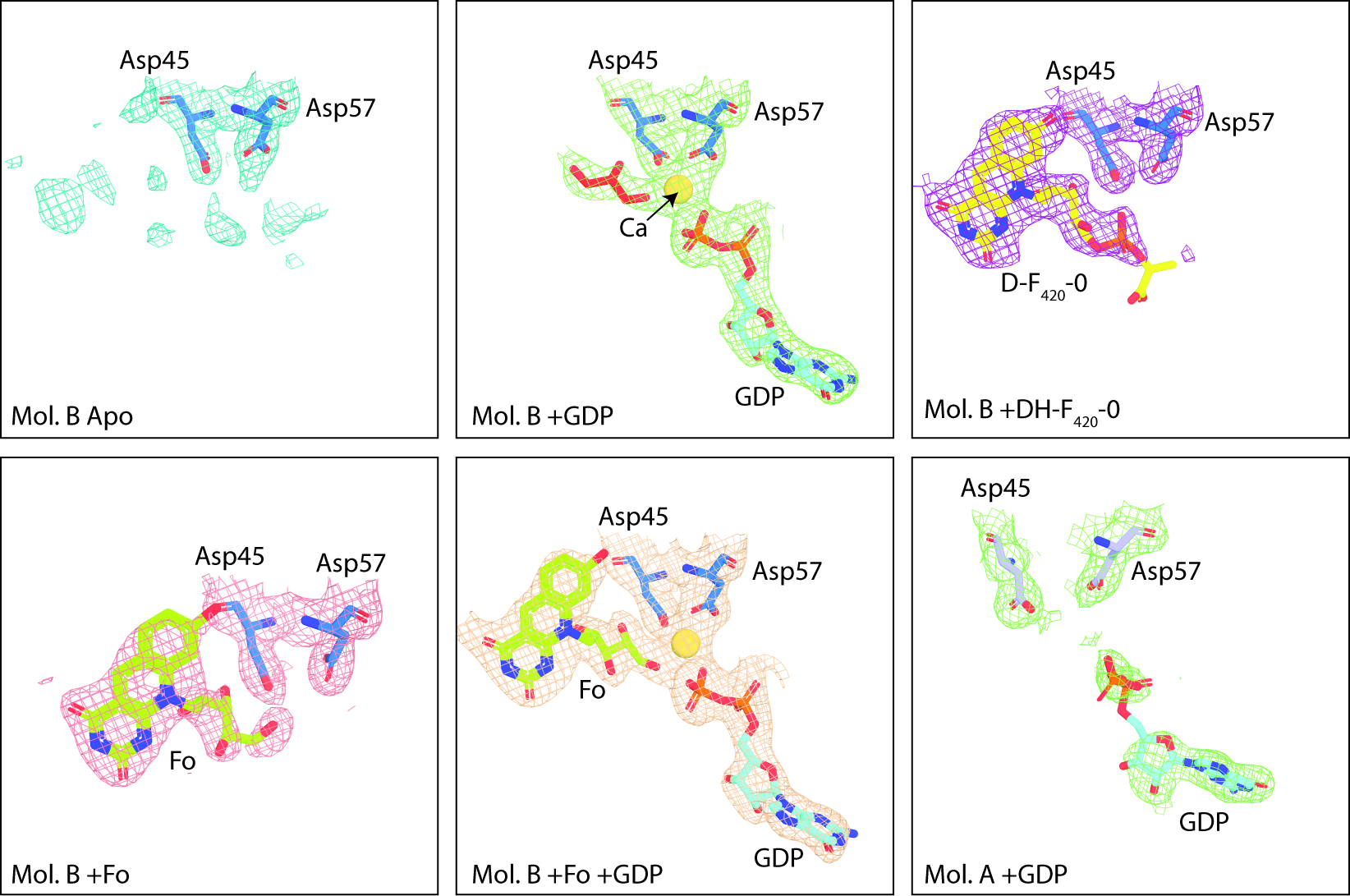

Supplement: FIG S3 [file mSystems.00389-20-sf003.tif]

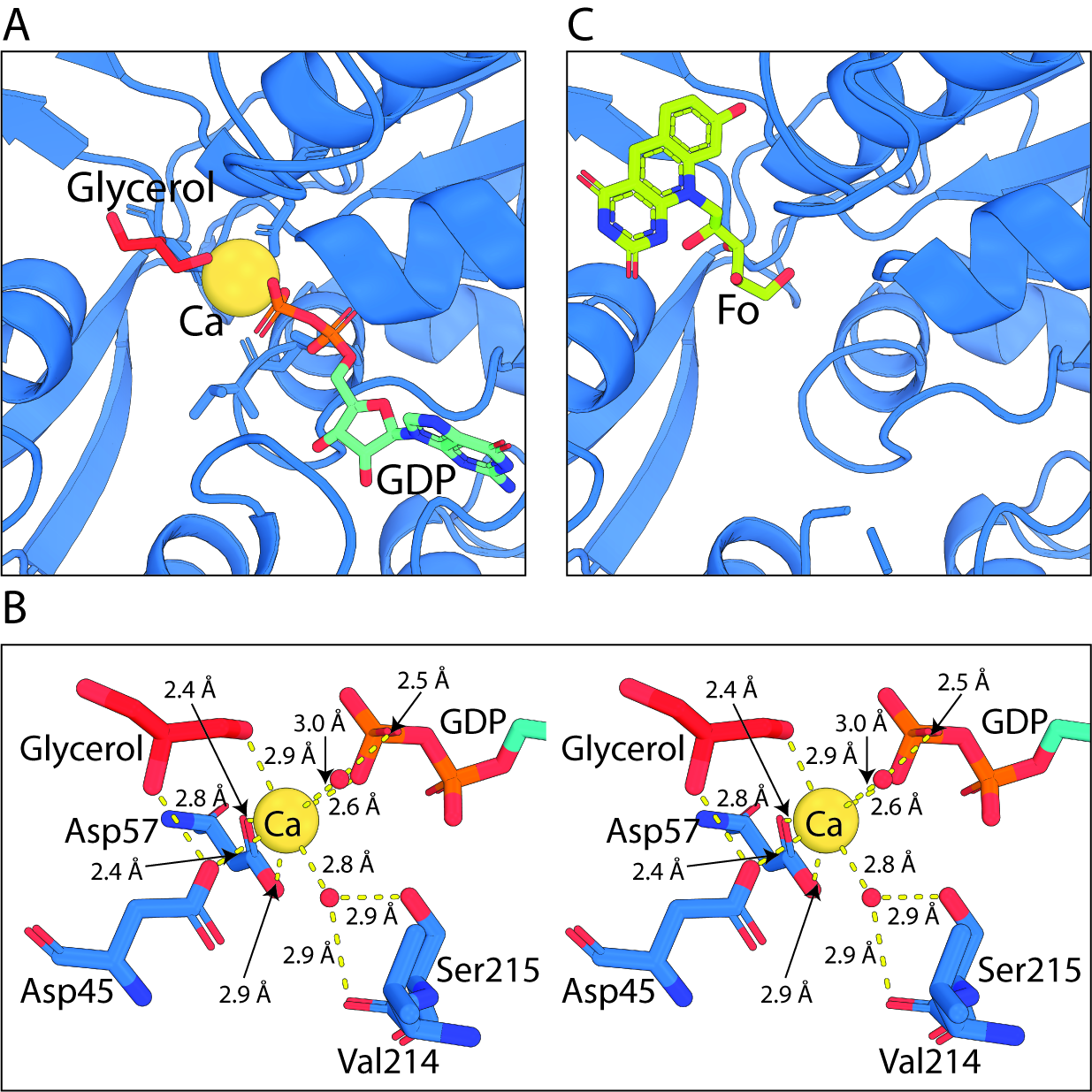

Supplement: FIG S4 [file mSystems.00389-20-sf004.tif]

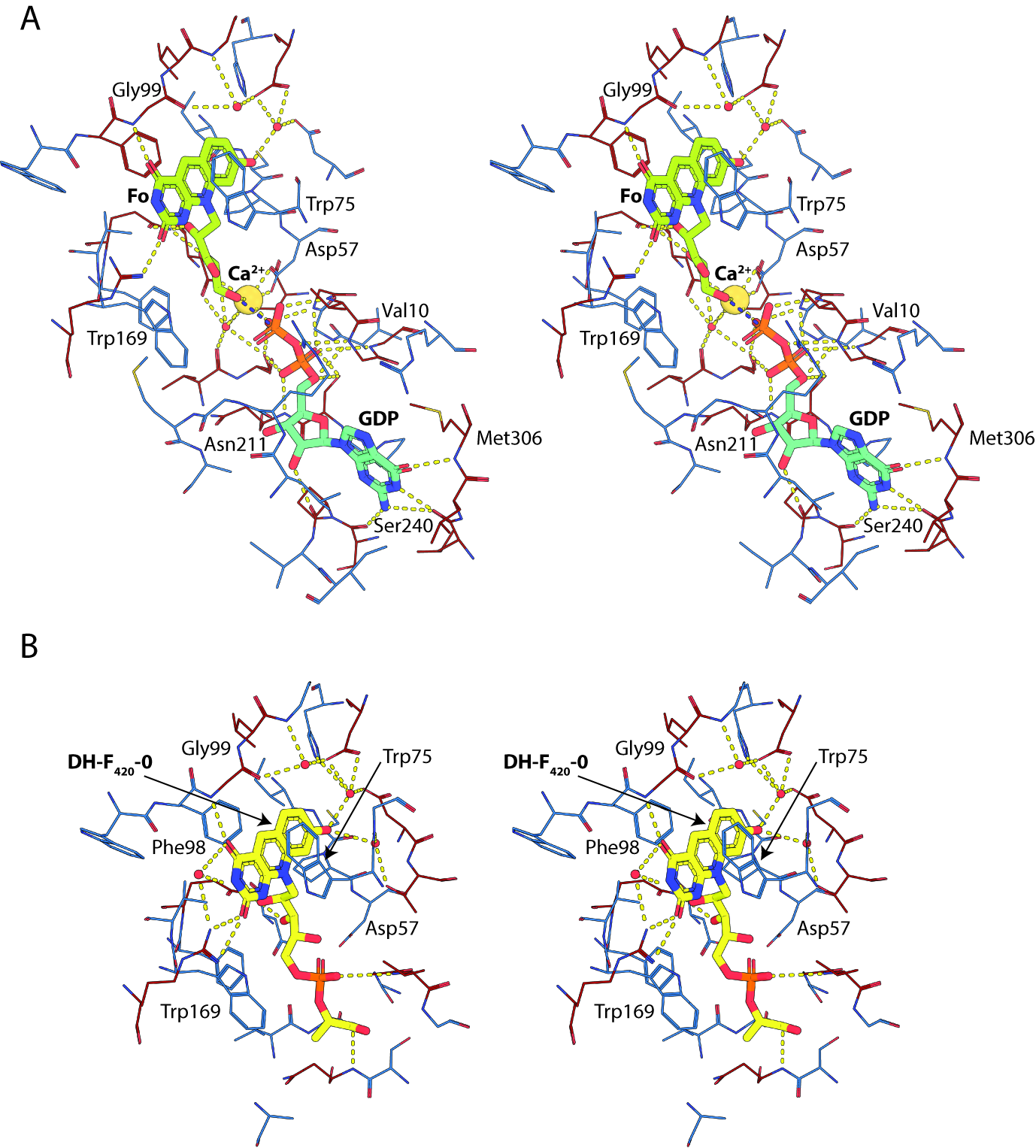

Supplement: FIG S5 [file mSystems.00389-20-sf005.tif]
